# Target attainment and population pharmacokinetics of flucloxacillin in critically ill patients: a multicenter study

**DOI:** 10.1186/s13054-023-04353-5

**Published:** 2023-03-03

**Authors:** Sjoerd D. Meenks, Nieko Punt, Jos L. M. L. le Noble, Norbert A. Foudraine, Kees Neef, Paddy K. C. Janssen

**Affiliations:** 1 Department of Clinical Pharmacy and Toxicology, Maastricht University Medical Center+, P.O. Box 5800, 6202 AZ Maastricht, The Netherlands; 2 Department of Clinical Pharmacy, Catharina Hospital Eindhoven, P.O. Box 1350, 5602 ZA Eindhoven, The Netherlands; 3 Department of Hospital Pharmacy, VieCuri Medical Center, 5900 BX Venlo, The Netherlands; 4 Medimatics, 6229 HR Maastricht, The Netherlands; 5 University Medical Center Groningen, Department of Clinical Pharmacy and Pharmacology, University of Groningen, Groningen, The Netherlands; 6 Department of Intensive Care, VieCuri Medical Center, 5900 BX Venlo, The Netherlands; 7 Department of Pharmacology and Toxicology, P.O. Box 616, 6200 MD Maastricht, The Netherlands

**Keywords:** Flucloxacillin, Pharmacokinetics, Critically ill, PK/PD target attainment, Free or unbound concentration

## Abstract

**Purpose:**

Insufficient antimicrobial exposure has been associated with worse clinical outcomes. Reportedly**,** flucloxacillin target attainment in critically ill patients was heterogeneous considering the study population selection and reported target attainment percentages. Therefore, we assessed flucloxacillin population pharmacokinetics (PK) and target attainment in critically ill patients.

**Methods:**

This prospective, multicenter, observational study was conducted from May 2017 to October 2019 and included adult, critically ill patients administered flucloxacillin intravenously. Patients with renal replacement therapy or liver cirrhosis were excluded. We developed and qualified an integrated PK model for total and unbound serum flucloxacillin concentrations. Monte Carlo dosing simulations were performed to assess target attainment. The unbound target serum concentration was four times the minimum inhibitory concentration (MIC) for ≥ 50% of the dosing interval (ƒT_>4xMIC_ ≥ 50%).

**Results:**

We analyzed 163 blood samples from 31 patients. A one-compartment model with linear plasma protein binding was selected as most appropriate. Dosing simulations revealed 26% ƒT_>2 mg/L_ ≥ 50% following continuous infusion of 12 g flucloxacillin and 51% ƒT_>2 mg/L_ ≥ 50% for 24 g.

**Conclusion:**

Based on our dosing simulations, standard flucloxacillin daily doses of up to 12 g may substantially enhance the risk of underdosing in critically ill patients. Prospective validation of these model predictions is needed.

**Supplementary Information:**

The online version contains supplementary material available at 10.1186/s13054-023-04353-5.

## Introduction

Despite the application of guideline-concordant antimicrobial therapy, severe infections still account for high mortality rates among critically ill patients [1, 2]. Insufficient antibiotic exposure or failure to attain the pharmacokinetic/pharmacodynamic (PK/PD) target has been associated with worse clinical outcomes [3–5]. However, adequate antibiotic dosing in critically ill patients is extremely complex, owing to pathophysiological changes and reduced antibiotic susceptibility to the pathogen [6, 7]. PK/PD target attainment of up to 60% has been reported for beta-lactam antibiotics in critically ill patients [7, 8].

Flucloxacillin is widely used to treat infections caused by Gram-positive bacteria [9]. In critically ill patients, flucloxacillin exhibits variable plasma protein binding, ranging from 28 to 97% [10, 11]. Maximal in vivo bactericidal activity of flucloxacillin and suppression of antimicrobial resistance can be expected when unbound serum concentrations exceed four times the minimum inhibitory concentration (MIC) for 50 to 100% of the dosing interval (ƒT_>4×MIC_ = 50–100%) [12–15]. These high concentrations are required to treat more resistant pathogens and facilitate penetration of sufficient unbound flucloxacillin to the infectious extravascular regions in critically ill patients [2, 4, 13–18].

Previous studies assessing PK/PD target attainment of unbound flucloxacillin in critically ill patients were heterogeneous, considering study population selection and reported target attainment percentages [10, 11, 19–21]. Two studies reported over 99.9% target attainment for daily doses up to 12 g [11, 20], whereas others indicated only 26–91% target attainment [10, 19, 21]. Previous study populations consisted of patients with serum hypoalbuminemia (≤ 32 g/L) [10, 20], or reported median estimated glomerular filtration rates (eGFRs) of at least 96–122 mL/min [10, 20, 21]. In addition, most previous studies have reported study population ages of up to only 59 years [10, 11, 20, 21], considered non-representative of critically ill patients [2, 22]. Moreover, the median non-coronavirus disease (COVID-19) age was 67 years in Dutch critically ill patients [2].

Considering the above-listed findings, we performed a population PK multicenter study in a study population with widely ranging eGFRs and serum albumin concentrations, approximately 67 years of age. The main objectives were to assess flucloxacillin population PK and determine a dosing strategy that maximizes PK/PD target attainment in critically ill patients based on dosing simulations.

## Methods

### Study design and population

This prospective, multicenter study was performed at the intensive care unit (ICU) of two hospitals in the Netherlands. VieCuri Medical Center Noord-Limburg, an in-patient non-university teaching hospital, and Maastricht University Medical Center+, an in-patient university teaching hospital. The study was conducted from May 2017 and October 2019. Flucloxacillin therapy and dosing were undertaken at the discretion of the clinician. In accordance with Dutch national and local guidelines, the vast majority of critically ill patients receive flucloxacillin therapy only following positive cultures for methicillin-sensitive *Staphylococcus aureus* (MSSA) or clinical suspicion for MSSA infection [23]. As part of standard and routine clinical care, blood sampling was performed for laboratory measurements at least every 24 h. The remains of these arterial blood samples were collected on flucloxacillin treatment days at random time points, related to flucloxacillin dosing. Total and unbound serum flucloxacillin concentrations were analyzed using a validated ultra-performance liquid chromatography-tandem mass spectrometry (UPLC-MS/MS) analysis [24, 25], and a validated ultrafiltration technique. Detailed information on the flucloxacillin bioanalysis and validation is available in Additional file 1. Adult, critically ill patients were eligible for study enrollment if they had received flucloxacillin intravenously during ICU admittance or ≤ 24 h before ICU admission. Patients were excluded if they received renal replacement therapy (RRT), suffered from liver cirrhosis, or objected to the use of their residual blood samples for clinical research. In the case of multiple ICU admissions, we only considered the first admittance for this study.

### Data collection

Demographic data were registered and collected for each patient from the electronic hospital information system, including flucloxacillin dose and administration details, age, sex, body weight, height, Acute Physiology and Chronic Health Evaluation (APACHE) II score, blood chemistry, and co-medication. Blood chemistry included serum creatinine and albumin levels. Albumin was routinely quantified by performing a Bromocresol Purple colorimetric assay.

### Population pharmacokinetic model development

The obtained PK data were analyzed using the Bayesian PK modeling software program, EDSIM++ version 2.04 (Mediware, Prague, Czech Republic) [26–29]. An integrated PK model for total and unbound flucloxacillin PK was developed using the KINPOP++ module. A stepwise approach was used for model building, resulting in a final PK model. Individual PK parameters were calculated by maximum a posteriori Bayesian fitting. The Bayesian fitting model used the measured serum flucloxacillin concentration, population-based PK parameters, and expected variability in each parameter to predict individual PK parameters. Detailed methodological information on PK model building and qualification is available in Additional file 1.

### Monte Carlo dosing simulations

The EUCAST database lacks information on the MIC distribution of flucloxacillin for MSSA [Eucast]. Therefore, PK/PD target attainment simulations were performed using the epidemiological cut-off (ECOFF) value of cloxacillin for MSSA (0.5 mg/L) [30]. It has been reported that MIC distributions of cloxacillin and flucloxacillin for MSSA are similar [31]. Monte Carlo dosing simulations were performed to predict target attainment at steady state in 1000 virtual patients. Continuous and intermittent dosing regimens were applied for daily doses ranging from 4 to 24 g [32, 33], using MicLab 2.70 (Medimatics, Maastricht, The Netherlands) [34, 35]. PK/PD targets were set at ƒT_>MIC_ ≥ 50%, ƒT_>MIC_ = 100%, ƒT_>4xMIC_ ≥ 50%, and ƒT_>4xMIC_ = 100%. Population PK parameters were assumed to be log-normally distributed (mean ± standard deviation [SD]). Intermittent flucloxacillin infusion duration was set at 0.5 h. MIC range was 0–4 mg/L, with MIC bins set to 0.0625 mg/L. The confidence interval for the distribution analysis was set at 95%. Covariance between model parameters was assumed to be absent.

### Statistical analysis

Statistical analyses were performed using IBM SPSS Statistics (version 24.0; IBM Corp, Armonk, NY, USA). Patient demographic information was presented using descriptive statistics. A Kolmogorov–Smirnov test was used to verify the normality of the distribution of continuous variables. Continuous variables are expressed as mean ± SD or median (25% to 75% interquartile range [IQR]), where appropriate. Discrete variables are expressed as counts and percentages.

## Results

### Patient and sample characteristics

Table 1 presents the demographic and clinical information of 31 patients. The median age was 69 years (interquartile range [IQR]: 54–76 years), and 18 were male (58%). The median total body weight was 76 kg (IQR: 64–85 kg). The mean APACHE II score was 21 ± 10, and 17 patients (55%) underwent mechanical ventilation. At ICU admission, the mean serum albumin level was 25.8 g/L, and the mean creatinine clearance (CL_cr_) (calculated using the CKD-EPI equation) was 68 ± 42 mL/min/1.73m^2^. Applied flucloxacillin daily doses ranged from 4 to 12 g, where 19 patients (61%) received 12 g of flucloxacillin. Flucloxacillin was administered intravenously for 15–30 min in 8 patients (26%), and by continuous infusion in 23 patients (74%). Detailed serum albumin and renal function characteristics of the patients are shown in Additional file 1: Table S1.

**Table 1:** Demographic and clinical characteristics of the study population

Characteristic	All patients (*n* = 31)
Male sex, %	58
Age, years	69 (54–76)
Height, cm, mean (SD)	172 (10)
ICU admission total body weight, kg	76 (64–85)
ICU admission BMI, kg/m^2^	25.3 (23.4–28.9)
*Admission details*	
APACHE II score, mean (SD)	21 (10)
Hospital admission duration, days	19 (9–33)
Hospital mortality, %	39
ICU admission duration, days	6 (2–17)
ICU mortality, %	26
Mechanical ventilation, %	55
Sepsis, %	48
Tertiary referral ICU, %	23
*Laboratory values*	
Albumin serum at ICU admission, g/L, mean (SD)	25.8 (8.5)
Albumin serum, g/L, mean (SD)	23.3 (8.2)
Creatinine serum at ICU admission, µmol/L	82 (63–159)
Creatinine serum, µmol/L	82 (66–222)
Creatinine clearance^a^ at ICU admission, mL/min/1.73m^2^, mean (SD)	68 (42)
Creatinine clearance^a^, mL/min/1.73m^2^, mean (SD)	66 (42)
*Flucloxacillin dose prescribedb*	
6 g per 24 h cont, %	24
9 g per 24 h cont, %	6
12 g per 24 h cont, %	44
1 g q6h, %	6
1 g q4h, %	9
2 g q4h, %	12

Characteristics of the study population at flucloxacillin sampling, unless stated otherwise. Values are expressed as median (interquartile range), unless stated otherwise. Percentages are rounded to whole numbers

*APACHE* Acute Physiology and Chronic Health Evaluation, *BMI* body mass index, *cont* continuous infusion, *CKD-EPI* Chronic Kidney Disease Epidemiology Collaboration, *ICU* intensive care unit, *q4h* 6 times daily, *q6h* 4 times daily, *SD* standard deviation

^a^Creatinine clearance was calculated using the CKD-EPI equation

^b^Three patients received two different flucloxacillin dosing regimens during ICU admission

In total, 163 blood samples were analyzed for total and unbound flucloxacillin concentrations, corresponding to a median of four samples per patient (IQR: 2–10). Measured total concentrations ranged from 1.3 to 668 mg/L, and unbound concentrations ranged from 0.6 to 137 mg/L. The median unbound fraction was 22% (IQR: 20–27), ranging from 6 to 73% in all analyzed blood samples.

### Population PK model development

Key steps in the population PK model development are shown in Table S2.

The one-compartment model with linear plasma protein binding was selected as the most appropriate base model (model 1), mainly based on an objective function value (OFV) of 961 and faster PK parameter convergence. Compared to model 1, adding eGFR as a covariate (model 4a; OFV = 887, *p* < 0.0001) or serum albumin (model 4b; OFV = 957, *p* = 0.0455) could improve the model. Compared to model 4a, combining both covariates could further improve the model (model 5; OFV 883; *p* = 0.0455), thereby resulting in the final population PK model.

Covariate analysis was performed using the following equations:

$$f_{{\text{u}}} = \, 0.{217 }* \, \left( {{\text{Alb }}/{ 21}.{2}} \right)^{ - ex}$$

where ƒ_u_ is the fraction unbound, Alb is the serum albumin concentration, and *ex* is the exponent for serum albumin. The population estimate of the unbound fraction was 0.217. The population estimate of serum albumin concentration was 21.2 g/L.

$${\text{CL}}_{{{\text{tot}}}} = \, f_{{\text{r}}} *{\text{ CL}}_{{{\text{cr}}}}$$

where CL_tot_ is the total drug clearance, ƒ_r_ is the effect size of creatinine clearance, and CL_cr_ is the serum creatinine clearance.

**Fig. 1:**
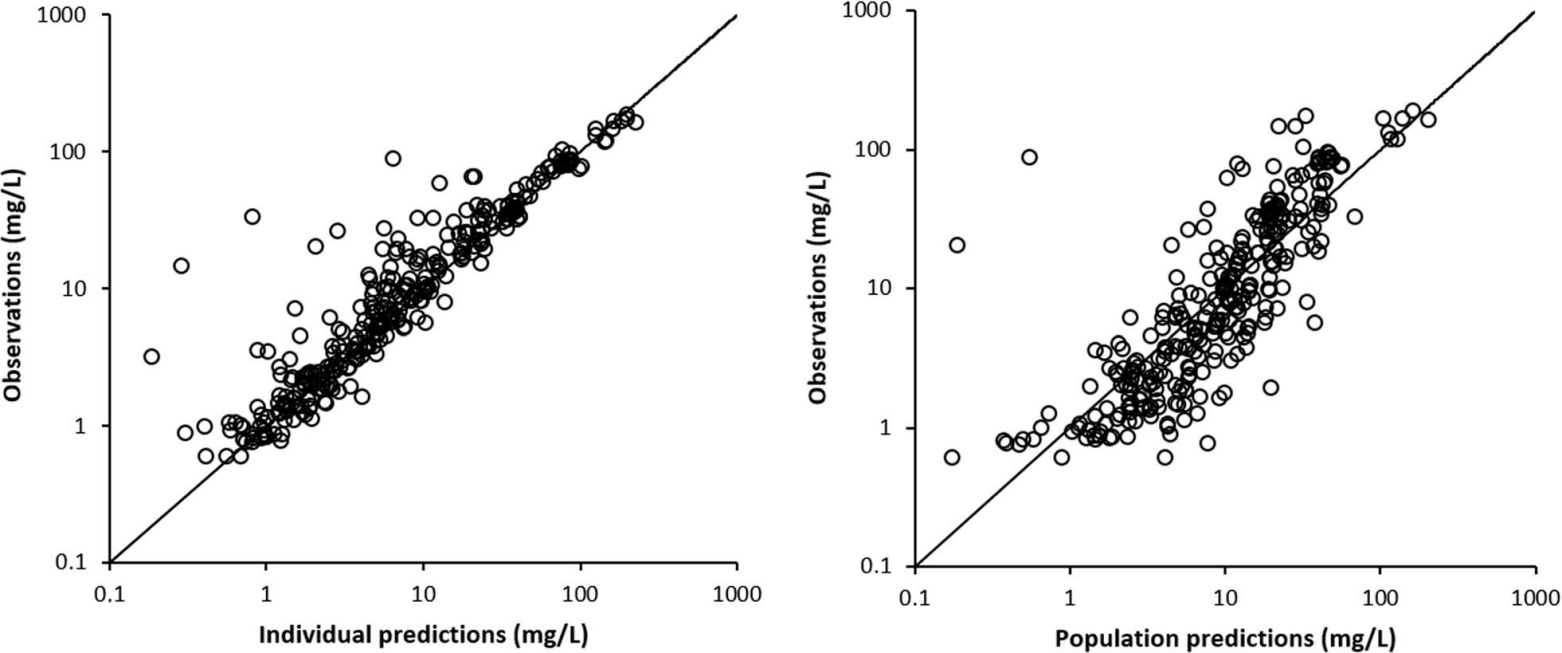
Goodness-of-fit plots of the final population PK model. Observed concentrations versus individual and population-predicted concentrations. Circles indicate observed data points, whereas the solid line represents the line of unity. *PK* pharmacokinetics

The final model bias was -27.1%, and precision was 53.3%. No systematic bias in model prediction was observed in the individual and population goodness-of-fit plots (Fig. 1). Final parameter estimates and bootstrap results are shown in Table 2. PK parameter predictions of the bootstrap analysis agreed with the parameter estimates of the final model.

**Table 2:** Parameter estimates, bootstrap medians, and confidence intervals

Parameter	Base model		Final model		Bootstrap of final model	
	Estimate, mean	RSE,%	Estimate, mean	RSE, %	Estimate, median	95% CI
CL, L/h/70kg^0.75^	52.8	23	–	–	–	–
ƒ_r_, CL/CL_cr_	–	–	19	13	19	12–27
V, L/70 kg	324	14	330	15	321	204–476
ƒ_u_, %	24	6	25	4.7	25	21–30
*ex*	-	-	0.67	16	0.63	0.29–1.14
Proportional error, total flucloxacillin, %	44	5	42	5.2	42	26–52
Proportional error, unbound flucloxacillin, %	35	5	35	5.2	35	25–42
IIV CL, %	127	–	–	–	-	–
IIV ƒ_r_, %	–	–	71	–	70	–
IIV V, %	79	–	84	–	80	–
IIV ƒ_u_, %	31	–	26	–	23	–
IIV *ex*, %	–	–	88	–	78	–

*CI* confidence interval, *CL* clearance, ƒ_r_ the unbound renal clearance of flucloxacillin divided by the creatinine clearance, *ƒ*_*u*_ fraction unbound, *IIV* inter-individual variability, *RSE* relative standard error, *ex* exponent for serum albumin, *V* volume of distribution

### Monte Carlo dosing simulations

**Fig. 2:**
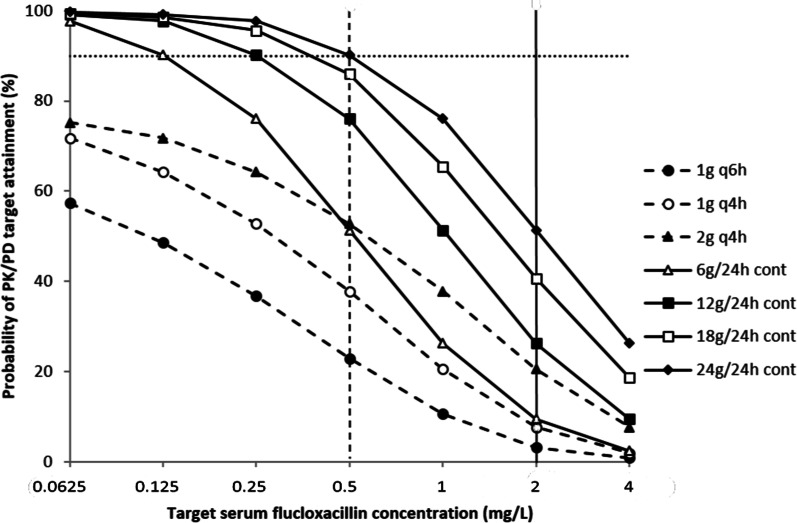
Probability of PK/PD target attainment for flucloxacillin at different dosing regimens and target unbound serum concentrations, based on Monte Carlo dosing simulations using the final PK model. PK/PD target attainment of flucloxacillin could be achieved when unbound serum concentrations exceed four times the MIC of the microorganism to be treated during at least 50% of the dosing interval (ƒT_>4xMIC_ ≥ 50%). The dashed vertical line represents the MIC breakpoint of flucloxacillin for MSSA (0.5 mg/L). The solid vertical line represents the target concentration of unbound flucloxacillin of four times the MIC of the microorganism to be treated in critically ill patients (2 mg/L). The horizontal dotted line represents a 90% probability of PK/PD target attainment. *cont* continuous infusion, *MSSA* methicillin-sensitive *Staphylococcus aureus*, *PD* pharmacodynamics, *PK* pharmacokinetics, *q4h* six times daily, *q6h* four times daily

Figure 2 presents the probability of PK/PD target attainment at steady state. The final PK model revealed 26% ƒT_>4xMIC_ ≥ 50% following daily continuous infusion of 12 g flucloxacillin and 51% ƒT_>4xMIC_ ≥ 50% following continuous infusion of 24 g. Dosing simulations with a PK/PD target set at ƒT_>4xMIC_ = 100% are demonstrated in Additional file 2: Fig. S1.

The percentages of PK/PD target attainment with the final PK model, as well as with the base, eGFR and albumin submodels, are provided in Additional file 1: Table S3.

## Discussion

In the present study, we describe the development of a population PK model for flucloxacillin in critically ill patients and consecutively PK/PD target attainment in this population, based on dosing simulations. The main study finding was that critically ill patients were at a considerable risk of underdosing when flucloxacillin was employed in standard daily doses of up to 12 g.

Dosing simulations revealed only 26% PK/PD target attainment (≥ 50% ƒT_>2 mg/L_) following daily continuous infusion of 12 g flucloxacillin. These results are inconsistent with findings of previous dosing simulation studies performed in critically ill patients [10, 11, 20]. Two studies reported ≥ 99.9% target attainment for 8 to 12 g per 24 h, with target serum concentrations of 2 to 2.5 mg/L [11, 20]. In addition, Jager et al*.* have reported 91% target attainment in patients with an eGFR of 33 mL/min and 71% for an eGFR of 153 mL/min with 2 g administered 6 times daily (q4h), accompanied by a PK/PD target of 100% ƒT_>0.5 mg/L_ [10]. However, our study results are in line with PK/PD target attainment as reported in two prospective, observational studies [19, 21]. Moser et al*.* have reported 26% target attainment (100% ƒT_>2 mg/L_) for 2 g administered 4 to 6 times daily; however, the authors also reported ‘optimal’ PK/PD target attainment of 90% when target serum concentrations were based on strain-specific MICs or 0.25 mg/L [19]. Wong et al*.* [21] have documented 52% target attainment (100% ƒT_>strain-specific MIC_) for 2 g q4h and 30% target attainment for 100% ƒT_>4 x strain-specific MIC_.

Several aspects could have contributed to differences in percentages of flucloxacillin PK/PD target attainment between our study and those reported previously [10, 11, 19–21], including (1) heterogeneity of the critically ill population, (2) complexity of plasma protein binding, and (3) appropriate selection of the target serum flucloxacillin concentration.

First, critically ill patients are known to exhibit considerable heterogeneity [7]. Previous studies have focused on critically ill subpopulations, complicating the comparison of study results [10, 20, 21]. Wallenburg et al*.* [11] have performed a dosing simulation study in a population most comparable to the present study population. Despite the older age of our study population, the calculated eGFR was comparable between both studies. In addition, non-renal drug clearance is generally preserved in elderly patients [36]. However, we detected a substantially reduced PK/PD target attainment, which may partly be explained by an elevated median flucloxacillin clearance of 77.5 L/h when compared with 37.5 L/h. In addition, the study population of Wallenburg et al*.* [11] consisted of 21% of patients who underwent continuous RRT and patients with liver cirrhosis may have been included, whereas these patients were excluded in our study. Furthermore, we noted a slightly elevated ƒ_u_, potentially resulting in increased non-renal clearance and tubular secretion. The PK model performance was improved by incorporating albumin and eGFR covariates, which is consistent with previous study results [10, 11]. No other significant model covariates were found to alter flucloxacillin PK, protein binding, and clearance. However, our study population presented a high body weight and BMI, along with an increased volume of distribution, and consisted of older patients [10, 11, 20]. These aspects may have contributed to the remaining proportional PK model error of up to 42%.

Second, plasma protein binding of flucloxacillin in critically ill patients remains complex [37–39]. Flucloxacillin and albumin concentrations reportedly impact protein binding and PK [10, 11, 40, 41]. However, these individual values may be difficult to interpret, for instance, due to both covalent and non-covalent bindings of flucloxacillin to plasma proteins or penicillin-induced pseudo-hypoalbuminemia [42–45]. The median observed ƒ_u_ in our study was 22%, which was slightly higher than the 7 to 19% reported in previous studies [10, 11, 19]. The observed broad ƒ_u_ range of 6–73% in our study is in line with previous studies [10, 11, 19]. Interestingly, the median serum albumin concentration in the present study was slightly higher than in most previous studies [10, 11, 19, 20]. However, the higher ƒ_u_ might be related to our older study population. For instance, plasma protein binding and flucloxacillin displacement from plasma proteins could be altered in older ICU patients owing to endogen molecules and polypharmacy [40, 46]. Inter-individual variance (IIV) on albumin or ƒ_u_ in our study was higher than that reported in other studies [10, 11, 19–21], mainly related to the exclusion of patients with serum albumin concentrations > 32 g/L in several previous studies [10, 20].

Third, target unbound serum flucloxacillin concentrations remain poorly defined [6, 47, 48]. An ECOFF value for flucloxacillin is lacking [30] but is stated to be similar to that of oxacillin and cloxacillin. However, cloxacillin ECOFF is 0.5 mg/L, and oxacillin ECOFF is 2 mg/L [30]. In the present study, we selected a target of 50% ƒT_>4x0.5 mg/L_, representing a target serum concentration of 2 mg/L; if a MIC of 2 mg/L had been selected, we would have attained even lower target attainment percentages. In addition, some studies mentioned target concentration selection based on strain-specific MICs from positive blood cultures [19, 21]. However, target concentration selection based on a single MIC determination has been deemed inappropriate and could be detrimental to patient therapy [48, 49]. First, routine clinical laboratories cannot accurately determine individual MICs owing to the inherent assay variation. Second, biological variation exists within a species even when there are no acquired resistance mechanisms [48]. Furthermore, we selected a serum target concentration of 4 times the ECOFF value [30]. Some previous studies reported improved clinical or microbiological cure for beta-lactam antibiotics when serum concentrations 2.1 to 5 times the MIC were achieved [12, 13, 15]. In critically ill patients, a higher incidence of more resistant pathogens is reported, and antibiotic tissue penetration may be impaired [2, 4, 13–18]. Therefore, to optimize antimicrobial efficacy and battle antimicrobial resistance, it is essential to eliminate all targeted pathogens, and target concentrations should be based on ECOFF values [30, 48, 49].

Our study has certain limitations. First, limited sampling of flucloxacillin (1–3 samples) was performed for most patients (74%), which could have potentially resulted in a suboptimal description of the individual PK and, consequently, the predicted population PK. Conversely, rich sampling data were available from 8 patients (26%), with up to 28 samples per patient. Additionally, a multicenter study was performed, and appropriate population PK model performance was demonstrated. Second, dosing simulations were performed; however, the collection of flucloxacillin concentration measurements from real patients would have been preferred. Unfortunately, the inclusion of large numbers of critically ill patients in PK studies can be challenging [33]. Third, patients suffering from liver cirrhosis or receiving RRT were excluded from the present study, restricting the current study results from representing the entire ICU population. Fourth, CL_cr_ was not actually measured, but we used the CKD-EPI equation to estimate CL_cr_. More accurate methods have been described in critically ill patients, such as calculating urine-to-plasma creatinine ratios [50]. Fifth, individual pathogen and MIC determination were not acquired, which could have aided the interpretation of appropriate flucloxacillin exposure.

Future research should focus on identifying efficacy and toxicity thresholds to maximize antimicrobial exposure and efficacy in critically ill patients [21]. Also, further research is required to assess which specific patients are at risk for flucloxacillin underexposure. For instance, underexposure may be related to age, APACHE II score or time since flucloxacillin treatment initiation. In addition, to comprehensively elucidate the in vivo equilibrium between protein-bound and unbound flucloxacillin, the complexity of flucloxacillin plasma protein binding needs to be further unraveled.

## Conclusion

Based on our dosing simulations, standard flucloxacillin daily doses of up to 12 g may substantially enhance the risk of underdosing in critically ill patients. Prospective validation of these model predictions is needed.

## Supplementary Information

**Additional file 1.** Supplementary e-Appendix.

**Additional file 2.** Figure S1.

## Data Availability

The data supporting the findings of the article are available on request by contacting the corresponding author.
