# Supplementary material for: Target attainment and population pharmacokinetics of flucloxacillin in critically ill patients: a multicenter study
Source: Crit Care. 2023 Mar 3;27:82. doi: 10.1186/s13054-023-04353-5 (PMC9982780; doi:10.1186/s13054-023-04353-5)
Supplement: Supplementary file 1 — Additional file 1. Supplementary e-Appendix. [file 13054_2023_4353_MOESM1_ESM.docx]

**Supplementary e-Appendix**

**Contents: Page number**

Bioanalysis of flucloxacillin in serum 2

Population pharmacokinetic model development 2

- Base model 2
- Covariate models 4
- Population pharmacokinetic model qualification 4

Table S1: Detailed serum albumin and renal function characteristics of the study population 5

Table S2: Key steps in population PK model development 6

Table S3: Percentages of PK/PD target attainment of flucloxacillin

at steady state, based on Monte Carlo dosing simulations 7

Figure S1: Probability of PK/PD target attainment for flucloxacillin at different dosing regimen

and target unbound serum concentrations, based on Monte Carlo dosing simulations 9

- Final model ƒT_>4xMIC_ = 100% (Fig. S1) 9

References Supplementary e-Appendix 10

**Bioanalysis of flucloxacillin in serum**

During routine clinical care, blood samples were centrifuged within 30 min from sampling and stored at 2 to 8^o^C. Residual samples were collected and stored for up to 2 weeks at -20°C until batch-wise analysis. The samples were analysed using a validated ultra-performance liquid chromatography-tandem mass spectrometry (UPLC-MS/MS) analysis [1, 2]. In addition, an ultrafiltration method was developed and validated. Bioanalysis was performed in the ISO 15189 certified clinical pharmaceutical laboratory at VieCuri Medical Centre [3].

Chromatographic analyses were performed using an Acquity UPLC H-class system equipped with a BEH C_18_ 50 × 2.1 mm 1.7 µm column and Xevo^®^ Triple Quadrupole Detector (Waters Corporation, Milford, MA, USA). The assay was linear for flucloxacillin concentrations up to 200 mg/L with a correlation coefficient (r) of 0.999 and regression coefficient (R^2^) of 0.998. Limit of quantification was 0.5 mg/L with a coefficient of variation (CV) of 4.5%. Within-assay variability was < 3.4% and between-assay variability was < 4.1%. The accuracy of the assay varied from 98.5 to 100.0%.

In addition to total flucloxacillin quantification, a method was developed to determine the protein unbound concentration. Fresh pooled plasma from non-critically ill patients was spiked to obtain total flucloxacillin concentrations of 4.0, 25 and 160 mg/L. First, in 6-fold, 0.5 to 0.7 mL of the plasma samples was centrifuged at 1500 *g* for 10 min at 25°C using a Centrifree^®^ Ultrafiltration Device with a 30,000 molecular weight cut-off (Merck Millipore, Tullagreen, Ireland). The ultrafiltrate was subsequently processed as a typical serum sample. Ultrafiltration accuracy was based on 95% flucloxacillin plasma protein binding in non-critically ill patients [4]. Ultrafiltration, accuracy was 98.0 to 101.2%, within-day variability was < 8.1%, and between-day variability was < 9.7%.

**Population pharmacokinetic model development**

*Base model*

Different potential base models were tested in order to select the most appropriate base model. Both one- and two-compartment models were tested as well as models with linear and non-linear plasma protein binding. Log-normal distribution was assumed for inter-individual PK variance. Standard deviations and / or 95% confidence intervals were calculated to enable comparison with published data.

In the Free PK mode, total concentrations were calculated by the following equation, both in case of linear and non-linear protein binding [5]: C_tot_ = C_u_ / ƒ_u_

In this equation: C_tot_ is the total flucloxacillin concentration, C_u_ is the unbound concentration, and ƒ_u_ is the unbound fraction. In case of linear plasma protein binding, protein binding was fixed, and therefore not influenced by serum albumin or serum flucloxacillin concentrations. In case of non-linear plasma protein binding, ƒ_u_ was variable, and calculated by the following equations [5]: C_b_ = (B_max_ * C_u_) / (K_d_ + C_u_); C_tot_ = C_u_ + C_b_; and ƒ_u_ = C_u_ / C_tot_.

In these equations: C_b_ is the bound flucloxacillin concentration, B_max_ is the maximum binding constant, and K_d_ is the dissociation constant.

PK parameters consisted of the unbound renal clearance of flucloxacillin divided by the creatinine clearance (ƒ_r_), central volume of distribution (V_c_) and, in case of linear protein binding, the fraction unbound (ƒ_u_), and in case of non-linear protein binding, both the maximum binding capacity (B_max_) and the dissociation constant (K_d_) [4]. In case of a two-compartment model, PK parameters also consisted of peripheral volume of distribution (V_p_) and intercompartmental clearance (Q). Regular allometric scaling was applied for the volumes of distribution, calculated in L/70 kg body weight and CL_tot_, calculated in L/h/70 kg^^0.75^ [6].

Population PK parameters were calculated using an iterative 2-stage Bayesian fitting procedure, based on an integrated analysis of unbound and total flucloxacillin serum concentrations, and plasma protein binding parameters [7]. The PK parameters for unbound flucloxacillin were estimated from the unbound and total flucloxacillin serum concentrations, and patient characteristics (age, sex, body weight, body height, serum creatinine). Residual variability, i.e. the difference between observed and model estimated concentrations, was modelled for the proportional error for the unbound and total flucloxacillin concentrations.

Performance of the different potential base models was evaluated by (1) the Objective Function Value (OFV), (2) the Akaike Information Criterion (AIC), (3) the bias (median prediction error) and precision (median absolute prediction error) of the estimated parameters, (4) the magnitude of residual variability, (5) goodness of fit plots, and (6) the convergence of the calculated PK parameters.

*Covariate models*

Tested continuous covariate data were serum albumin concentration, body weight, and estimated glomerular filtration rate (eGFR - calculated using the Chronic Kidney Disease Epidemiology Collaboration [CKD-EPI] equation). No categorical covariate data were evaluated. A power function or linear regression was applied, where appropriate. Reductions in OFV, AIC, inter individual variance (IIV) and residual variability, and improvement of the goodness of fit plots were used as a criterion for selection of relevant covariates, resulting in the final model. In addition, following the chi square distribution of the OFV, a *p* value of < 0.05 was considered as a statistically significant improvement of the PK model.

*Population pharmacokinetic model qualification*

Robustness of the final population covariate PK model was tested using a bootstrap analysis. The entire dataset was resampled and fitted to the model (n = 1000). Confidence interval for the PK parameters was set at 95%.

**Table S1 Detailed serum albumin and renal function characteristics of the study population**

| **Characteristic** | **All patients (*n* = 31)** |
| --- | --- |
| **Laboratory values at ICU admission** |  |
| Albumin serum, g/L, mean (SD) | 25.8 (8.5) |
| 10–19 g/L, % | 8 (26) |
| 20–34 g/L, % | 16 (52) |
| 35–55 g/L, % | 7 (23) |
| Creatinine serum, µmol/L | 82 (63–159) |
| Renal function |  |
| Creatinine clearance^a^, mL/min/1.73m^2^, mean (SD) | 68 (42) |
| < 15 mL/min/1.73m^2^, % | 7 |
| 15–29 mL/min/1.73m^2^, % | 16 |
| 30–59 mL/min/1.73m^2^, % | 29 |
| 60–89 mL/min/1.73m^2^, % | 13 |
| 90–119 mL/min/1.73m^2^, % | 19 |
| 120–149 mL/min/1.73m^2^, % | 16 |
| ≥ 150 mL/min/1.73m^2^, % | 0 |
| **Laboratory values at flucloxacillin sampling** |  |
| Albumin serum, g/L, mean (SD) | 23.3 (8.2) |
| 10–19 g/L, % | 9 (29) |
| 20–34 g/L, % | 15 (44) |
| 35–55 g/L, % | 7 (23) |
| Creatinine serum, µmol/L | 82 (66–222) |
| Renal function |  |
| Creatinine clearance^a^, mL/min/1.73m^2^_,_ mean (SD) | 66 (42) |
| < 15 mL/min/1.73m^2^, % | 13 |
| 15–29 mL/min/1.73m^2^, % | 10 |
| 30–59 mL/min/1.73m^2^, % | 23 |
| 60–89 mL/min/1.73m^2^, % | 26 |
| 90–119 mL/min/1.73m^2^, % | 19 |
| 120–149 mL/min/1.73m^2^, % | 7 |
| ≥ 150 mL/min/1.73m^2^, % | 3 |

Values are expressed as median (interquartile range), unless stated otherwise. Percentages are rounded to whole numbers

*CKD-EPI* Chronic Kidney Disease Epidemiology Collaboration, *ICU*, intensive care unit, *SD*, standard deviation

^a^ Creatinine clearance was calculated using the CKD-EPI equation

**Table S2 Key steps in population PK model development**

| **Model** | **OFV** | **Parameters** | **ΔOFV** | **PK parameter convergence, cycles** | ***p* value** |
| --- | --- | --- | --- | --- | --- |
| 1 (base model) | 961 | CL, V, ƒ_u_ | - | 23 | - |
| 2 | 1000 | CL, V_c_, ƒ_u_, Q, V_p_ | +39 to model 1 | 163 | - |
| 3 | 989 | CL, V, K_d_/B_max_ | - | 17 | - |
| 4a (eGFR model) | 887 | CL, V, ƒ_u_, eGFR | -74 to model 1 | 38 | < 0.0001 |
| 4b (albumin model) | 957 | CL, V, ƒ_u_, ALB, *ex* | -4 to model 1 | 32 | 0.0455 |
| 5 (final model) | 883 | CL, V, ƒ_u_, ALB, *ex*, eGFR | -4 to model 4a | 45 | 0.0455 |

Model 1 is the base model (one-compartment, linear plasma protein binding). Model 2 is a two-compartment model with linear plasma protein binding. Model 3 is a one-compartment model with non-linear plasma protein binding. Models 4a and 4b, eGFR or serum albumin was added as covariate. Model 5 is the final model, where both serum creatinine and serum albumin were added.

*ALB* serum albumin, *B_max_* maximum binding constant, *CL* clearance ([L/h/70kg]^0.75^), *eGFR* estimated glomerular filtration rate, ƒ*_u_*, fraction unbound, *K_d_* dissociation constant, *OFV* objective function value, *Q* intercompartmental flow rate, *ex* exponent for serum albumin, *V* volume of distribution (L/70kg) *V_c_* volume of distribution of the central compartment (L/70kg), *V_p_* volume of distribution of the peripheral compartment (L/70kg)

**Table S3 Percentages of PK/PD target attainment of flucloxacillin at steady state, based on Monte Carlo dosing simulations**

| **PK model** | **PK/PD target** |  | **PK/PD target attainment, %** | | | | | | |
| --- | --- | --- | --- | --- | --- | --- | --- | --- | --- |
|  |  |  | 1 g q6h | 1 g q4h | 2 g q4h | 6 g/24h cont | 12 g/24h cont | 18 g/24h cont | 24 g/24h cont |
| **Base model** | 50% ƒT_>MIC_ |  | 87 | 95 | 98 | 99 | 100 | 100 | 100 |
|  | 50% ƒT_>4xMIC_ |  | 42 | 64 | 86 | 68 | 92 | 98 | 99 |
|  | 100% ƒT_>MIC_ |  | 57 | 75 | 84 | 98 | 100 | 100 | 100 |
|  | 100% ƒT_>4xMIC_ |  | 20 | 38 | 59 | 61 | 88 | 96 | 98 |
| **eGFR model** | 50% ƒT_>MIC_ |  | 29 | 45 | 57 | 61 | 84 | 91 | 95 |
|  | 50% ƒT_>4xMIC_ |  | 4 | 11 | 27 | 13 | 36 | 50 | 61 |
|  | 100% ƒT_>MIC_ |  | 14 | 23 | 35 | 60 | 83 | 92 | 96 |
|  | 100% ƒT_>4xMIC_ |  | 1 | 6 | 14 | 14 | 33 | 48 | 60 |
| **Albumin model** | 50% ƒT_>MIC_ |  | 77 | 87 | 93 | 94 | 98 | 99 | 99 |
|  | 50% ƒT_>4xMIC_ |  | 43 | 58 | 76 | 63 | 84 | 92 | 94 |
|  | 100% ƒT_>MIC_ |  | 57 | 75 | 83 | 95 | 99 | 99 | 100 |
|  | 100% ƒT_>4xMIC_ |  | 20 | 37 | 59 | 56 | 82 | 91 | 95 |
| **Final model** | 50% ƒT_>MIC_ |  | 23 | 38 | 53 | 51 | 76 | 86 | 90 |
|  | 50% ƒT_>4xMIC_ |  | 3 | 8 | 20 | 9 | 26 | 41 | 51 |
|  | 100% ƒT_>MIC_ |  | 11 | 20 | 30 | 51 | 76 | 86 | 90 |
|  | 100% ƒT_>4xMIC_ |  | 2 | 4 | 11 | 9 | 26 | 41 | 51 |

ƒ*T_>MIC_* unbound flucloxacillin serum concentrations exceed the MIC of the microorganism to be treated during 50% or 100% of the dosing interval, *cont* continuous infusion, *MIC* minimum inhibitory concentration, *q4h* 6 times daily, *q6h* 4 times daily

**Fig. S1**


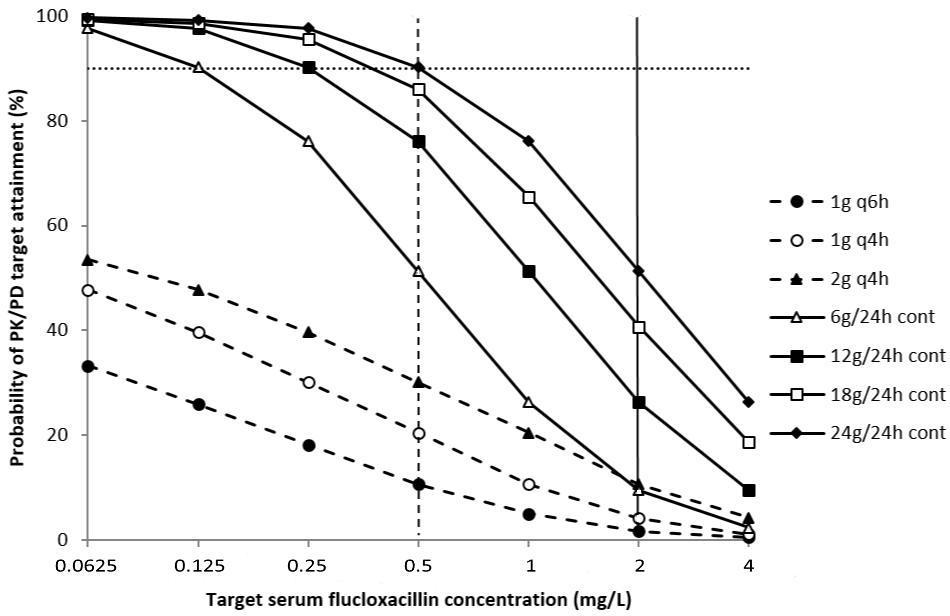


**Fig. S1** Probability of PK/PD target attainment for flucloxacillin at different dosing regimen and target unbound serum concentrations, when the PK/PD target would be set at ƒT_>4xMIC_ = 100%^a^. The dashed vertical line represents the MIC breakpoint of flucloxacillin for MSSA (0.5 mg/L). The solid vertical line represents the target concentration of unbound flucloxacillin of 4 times the MIC of the microorganism to be treated, in critically ill patients (2 mg/L). The horizontal dotted line represents 90% probability of PK/PD target attainment. *cont* continuous infusion, *q4h* 6 times daily, *q6h* 4 times daily.

^a^ ƒ*T_>4xMIC_ = 100%,* unbound flucloxacillin serum concentrations exceed 4 times the MIC of the microorganism to be treated during 100% of the dosing interval.

**References Supplementary e-Appendix**

1. EDQM guideline (2020) Validation of Analytical Procedures: PA/PH/OMCL (13) 82 R5. https://www.edqm.eu/. Accessed 07 December 2022.
2. ICH guidelines (2005) Validation of Analytical Procedures: Tekst and Methodology Q2(R1). https://www.ich.org/page/quality-guidelines.Accessed 07 December 2022.
3. [ISO (2012) ISO 15189:2012 - Medical laboratories — Requirements for quality and competence](https://www.iso.org/standard/56115.html). https://www.iso.org/standard/56115.html. Accessed 07 December 2022.
4. Bowmed Ibisqus Limited (2021) Product information Floxapen powder for solution for injection. www.medicines.org.uk. Accessed 6 September 2022.
5. Toutain PL, Bousquet-Melou A (2002) Free drug fraction vs free drug concentration: a matter of frequent confusion. J Vet Pharmacol Ther. 25(6): 460-463. <https://doi.org/10.1046/j.1365-2885.2002.00442.x>
6. Huang Q, Riviere JE (2014) The application of allometric scaling principles to predict pharmacokinetic parameters across species. Expert Opin Drug Metab Toxicol. 10(9): 1241-1253. <https://doi.org/10.1517/17425255.2014.934671>
7. Bonate PL (2001) A brief introduction to Monte Carlo simulation. [Clin Pharmacokinet](https://www.ncbi.nlm.nih.gov/pubmed/?term=Bonate+2001%2C+A+brief+introduction+to+monte+carlo+simulation%2C+Clin+Pharmacokinet). 40(1): 15-22. <https://doi.org/10.2165/00003088-200140010-00002>
