# Supplementary figures and images for: Target attainment and population pharmacokinetics of flucloxacillin in critically ill patients: a multicenter study
Source: Crit Care. 2023 Mar 3;27:82. doi: 10.1186/s13054-023-04353-5 (PMC9982780; doi:10.1186/s13054-023-04353-5)

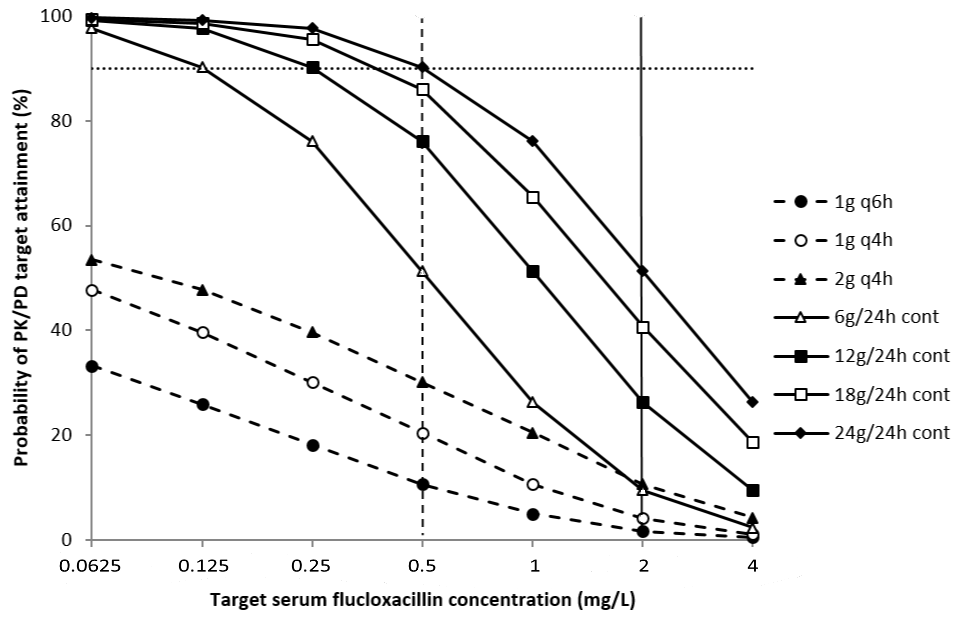

Supplement: Supplementary file 2 — Additional file 2. Figure S1. [file 13054_2023_4353_MOESM2_ESM.tiff]
